# Proteasome and Neuroprotective Effect of Hyperbaric Oxygen Preconditioning in Experimental Global Cerebral Ischemia in Rats

**DOI:** 10.3389/fneur.2022.812581

**Published:** 2022-02-17

**Authors:** Robert P. Ostrowski, Emanuela Pucko, Ewa Matyja

**Affiliations:** Department of Experimental and Clinical Neuropathology, Mossakowski Medical Research Institute, Polish Academy of Sciences, Warsaw, Poland

**Keywords:** proteasome, MG132, Bim, PUMA, nestin, cerebral ischemia, preconditioning, hyperbaric oxygen

## Abstract

**Objectives:**

We investigated the involvement of the proteasome in the mechanism of preconditioning with hyperbaric oxygen (HBO-PC).

**Methods:**

The experiments were performed on male Wistar rats subjected to a transient global cerebral ischemia of 5 min duration (2-vessel occlusion model) and preconditioned or not with HBO for 5 preceding days (1 h HBO at 2.5 atmosphere absolute [ATA] daily). In subgroups of preconditioned rats, the proteasome inhibitor MG132 was administered 30 min prior to each preconditioning session. Twenty-four hours and 7 days post-ischemia, after neurobehavioral assessment, the brains were collected and evaluated for morphological changes and quantitative immunohistochemistry of cell markers and apoptosis-related proteins.

**Results:**

We observed reduced damage of CA1 pyramidal cells in the HBO preconditioned group only at 7 days post-ischemia. However, both at early (24 h) and later (7 days) time points, HBO-PC enhanced the tissue expression of 20S core particle of the proteasome and of the nestin, diminished astroglial reactivity, and reduced p53, rabbit anti-p53 upregulated modulator of apoptosis (PUMA), and rabbit anti-B cell lymphoma-2 interacting mediator of cell death (Bim) expressions in the hippocampus and cerebral cortex. HBO-PC also improved T-maze performance at 7 days. Proteasome inhibitor abolished the beneficial effects of HBO-PC on post-ischemic neuronal injury and functional impairment and reduced the ischemic alterations in the expression of investigated proteins.

**Significance:**

Preconditioning with hyperbaric oxygen-induced brain protection against severe ischemic brain insult appears to involve the proteasome, which can be linked to a depletion of apoptotic proteins and improved regenerative potential.

## Introduction

It has been postulated that hyperbaric oxygenation can be used as a preconditioning modality to increase the resistance of neurons to ischemia. It can be used prior to major medical procedures that carry a risk of cerebral ischemia and, in general, when a brain injury is anticipated (1, 2). Hyperbaric oxygen preconditioning (HBO-PC) has been shown to provide neuroprotection in several models of cerebral ischemia (3–5). However it has not been determined whether HBO-PC is effective against very severe global brain ischemia, in which systemic blood pressure is reduced to 30 mmHg, such as in the modified two vessel occlusion (2VO) cerebral ischemic model (6). Moreover, the role of proteasomes in mechanisms of HBO-PC has not been as yet investigated. The involvement of the 26S proteasome, composed of one 20S proteasome core particle structure and two 19S regulatory caps, has been so far revealed for the rapid hypoxic preconditioning in neuronal cell cultures (7). One of the HBO-PC mechanisms possibly at play could be a depletion of cell death proteins. Such depletion could be mediated by the ubiquitin-proteasome system that is activated upon HBO-PC. In addition, regenerative capabilities of neural cells might become also be enhanced following HBO-PC. However, whether this effect depends on proteasomes has not been determined. Therefore, we proposed this study to evaluate the effect of the HBO-PC on ischemia-reperfusion brain injury and to examine the effect of proteasome inhibition on the preconditioning mechanism. We have therefore hypothesized that proteasome inhibition applied before each preconditioning session will abolish a preconditioning effect. Concomitantly, changes in tissue expression of 20S core particle of 26S proteasome and those of nestin and proteins involved in cell death mechanisms were investigated in the brain across all groups.

## Materials and Methods

### Experimental Groups

The study was performed on 40 male Wistar rats (provided by Medical Research Centre Animal Facility) weighing 280–350 g, randomly allocated to the following groups: naive control, sham surgery, (2VO) untreated, 2VO preconditioned with HBO (HBO-PC+2VO), and 2VO preconditioned with HBO and receiving the proteasome inhibitor MG132, injected intraperitoneal injection (i.p.) at a dose of 1 mg/kg, at 30 min prior to each HBO session (MG132+HBO-PC+2VO group). HBO-PC procedure involved 100% oxygen at 2.5 ATA for 1 h daily within 5 consecutive days out of which the last session started at 24 h before ischemia. The compressions and decompressions were performed at rates of 5 psi/min, by means of an RSI B11 research hyperbaric chamber (Reimers Systems, Inc., Alexandria, VA, USA). The proteasome inhibitor MG132 (Sigma-Aldrich, Darmstadt, Germany) was dissolved in 0.1% dimethyl sulfoxide (DMSO) in 0.9% NaCl.

### Global Cerebral Ischemia Model

Two-vessel occlusion was performed to induce global cerebral ischemia in mechanically ventilated rats. The surgeries were performed under ketamine (100 mg/kg b.w.) and xylazine (10 mg/kg b.w.) anesthesia (i.p.). Arterial blood pressure from the femoral artery catheter was continuously monitored by means of a pressure transducer connected to a BP-1 Pressure Monitor (WPI, Inc., Hitchin, United Kingdom).

Transient global cerebral ischemia was induced by clamping both common carotid arteries for 5 min while maintaining a profound hypotensive state, due to lowering mean arterial blood pressure (MABP) to 30–35 mmHg through exsanguination into a heparinized syringe (8). During surgeries and in the immediate post-operative period, the temperature was maintained at the level of 37 ± 0.5°C. The experiments involving laboratory animals complied with the Guide for the Care and Use of Laboratory Animals (ed. 2011), conformed to the Directive 2010/63/EU, and were approved by the Local Ethical Committee.

### Neurobehavioral Tests

T-maze testing was done at 7 days after surgery (9), while neuroscoring and mortality were assessed daily, by an experimenter blinded to the type of surgery and treatment. T-maze results were expressed as a percent of spontaneous alternations with respect to 50% reference (8). The modified Garcia score was used to evaluate sensorimotor deficits and included the assessment of spontaneous activity; symmetry in the movement of four limbs; forepaw outstretching; climbing; body proprioception; response to vibrissae touch; and beam balance (9).

### Histology Studies

At 24 h or on the 7th day after experimental ischemia, the animals were anesthetized with ketamine/xylazine and perfused through the ascending aorta with phosphate-buffered formalin. Following the perfusion, the brains were collected from the skulls and immersed in the same fixative overnight. The specimens were dehydrated in ethanol gradient and embedded in paraffin blocks. Brain sections 8 μm thick, encompassing dorsal hippocampus, were cut with SM2000R Microtome (Leica Microsystems, Nussloch, Germany) between 3.2 and 4.2 mm posterior to bregma and collected on the Silane-Prep glass slides (Sigma-Aldrich, St. Louis, MO, USA). Upon immunohistochemistry procedures, the specimens were deparaffinized with xylenes, preboiled in the citric buffer (pH 6.0), and incubated with human serum (1:50) for 0.5 h at room temperature (RT). The following primary antibodies were used for subsequent immunochemical stainings: rabbit anti-20S proteasome β1 (1:300, SCBT, Santa Cruz, CA, USA); rabbit anti-p53 (1:50, SCBT); rabbit anti-p53 upregulated modulator of apoptosis (PUMA) (1:300, SCBT); rabbit anti-B cell lymphoma-2 interacting mediator of cell death (Bim) (1:300, Enzo Life Sciences AG, Lausen, Switzerland); rabbit anti-nestin (1:50, Sigma-Aldrich, Schnelldorf, Germany) mouse anti-NeuN (1:100, Millipore, Temecula, CA, USA), and rabbit anti- glial fibrillary acidic protein (GFAP; 1:2,000, Dako, Glostrup, Denmark). Brain sections were incubated with the antibodies in serum for 1 h at RT, then washed and incubated with secondary biotin-conjugated antibodies for 0.5 h at RT. The immunochemical stain was developed with Streptavidin Peroxidase and 3,3′-Diaminobenzidine (DAB) chromogen (Dako, Carpinteria, CA, USA). Immunostained sections were counterstained with hematoxylin, then dehydrated, cleared in xylenes, and coverslipped with Kanadabalsam (Roth, Karlsruhe, Germany) (9, 10).

Klüver-Barrera staining and cell count were performed to evaluate the numbers of surviving pyramidal cells in the CA1 sector of the hippocampus (9). The immunostained brain sections were observed under Nikon E800 light microscope and digital camera connected to the PC computer equipped with NIKON software (Nikon Corporation, Tokyo, Japan). Regions of interest were photographed under ×40, ×100, and ×200 magnifications. Cell counts were done manually from images in four brains per group, marking immunoreactive cells using Image J software by experimenters blinded to the group ID and type of treatment (11). In brief, two slides with two brain sections on each were used for each brain. Six visual fields of the cerebral cortex were photographed in each section which resulted in 24 photographs from each brain and 96 per each group, as earlier described (9).

### Statistical Approach

Statistical analysis was conducted by means of parametric ANOVA or Kruskal-Wallis ANOVA, followed by Student-Newman-Keuls (SNK) test. A chi-square test was applied to analyze the mortality data (SigmaPlot 12.0). Probability level *p* < 0.05 was considered significant.

## Results

**Figure 1:**
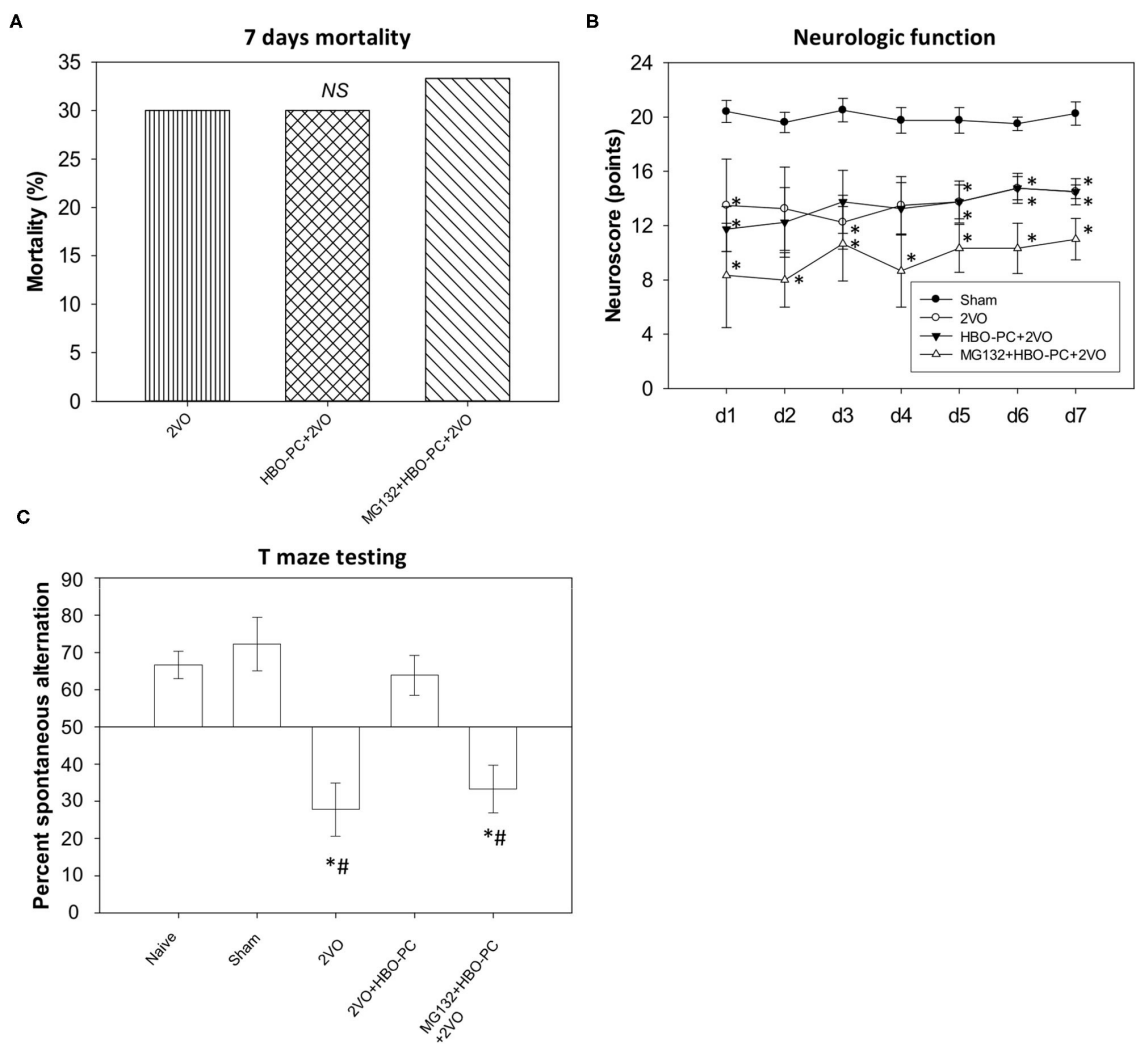
Mortality and neurobehavior after global brain ischemia. **(A)** Seven day mortality. An equivalent level of mortality was noted in post-ischemia of all groups **(B)** neuroscores. Equivalent reductions of neurologic scores were seen in all groups after ischemia. *p < 0.05 vs. sham; *n* = 4. **(C)** T-maze testing. Increased percent alternations between T-maze arms were detected in the preconditioned group; *p < 0.05 vs. sham; #*p* < 0.05 vs. HBO-PC+2VO; *n* = 4. 2VO, two vessel occlusion; HBO-PC, preconditioning with hyperbaric oxygen.

Seven-day mortality equaled 30% both in the non-preconditioned group and in the HBO preconditioned group (Figure 1A), while 33.3% mortality was noted in the MG132 group. The chi-square test confirmed that mortality levels were not significantly different between groups.

### Neurologic Function

All ischemic groups showed equivalent sensorimotor neuroscores, although reduced, as compared to sham group scores (Figure 1B).

### T-Maze Test Performance

In contrast, the HBO preconditioned rats showed a significantly higher percentage of spontaneous alternations between maze arms as compared with non-preconditioned rats at 7 days after ischemia (Figure 1C). However, the combination of MG132 and HBO-PC resulted in the deterioration of T-maze scores.

### Klüver-Barrera Staining Results and Cell Count

**Figure 2:**
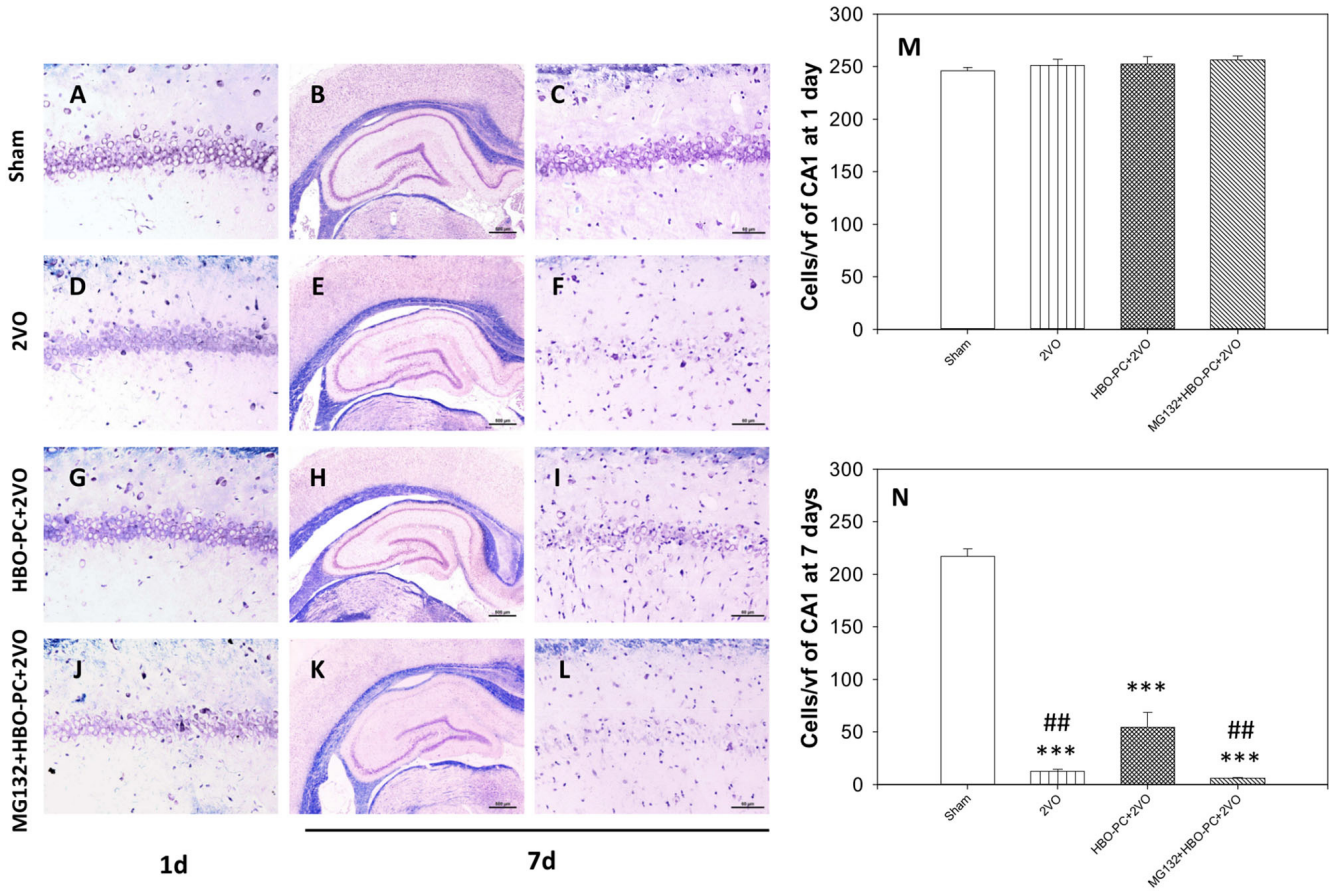
**(A–L)** Klüver-Barrera stain. HBO-PC ameliorated cell loss in the CA1 zone and proteasome inhibitor countered this effect. Scale bar = 60 μm. **(M,N)** Hippocampal cell count at 1 and 7 days after ischemia; ***p < 0.001 vs. sham; ##*p* < 0.01 vs. HBO-PC; *n* = 4. HBO-PC, preconditioning with hyperbaric oxygen.

At 24 h post-ischemia or sham surgery, well-preserved pyramidal neurons of CA1 were seen (Figures 2A,D,G,J). At 7 days after ischemia, light microscopic observations revealed a profound cell loss in the CA1 region (Figures 2E,F) as compared to the sham group (Figures 2B,C). However, substantial amelioration of post-ischemic CA1 neuronal damage was observed in the HBO-preconditioned group (Figures 2H,I). In contrast, conspicuous neuronal loss was found again on day 7 after ischemia, in HBO preconditioned group that received MG132 (Figures 2K,L). At 24 h after ischemia, cell count revealed equivalent numbers of CA1 pyramidal cells in all groups (Figure 2M). On day 7, the number of neurons in the post-ischemic CA1 hippocampal region of non-preconditioned rats was equal only to 5.7% of that in sham surgery control, whereas HBO-PC 4.3-fold increased the number of CA1 cells surviving 7 days after ischemia (Figure 2N). Statistical analysis showed a significant reduction in the number of CA1 neurons in rats pretreated with MG132 prior to preconditioning, as compared to the HBO-PC+2VO group.

### HBO-PC Effect on NeuN-Positive Cells

**Figure 3:**
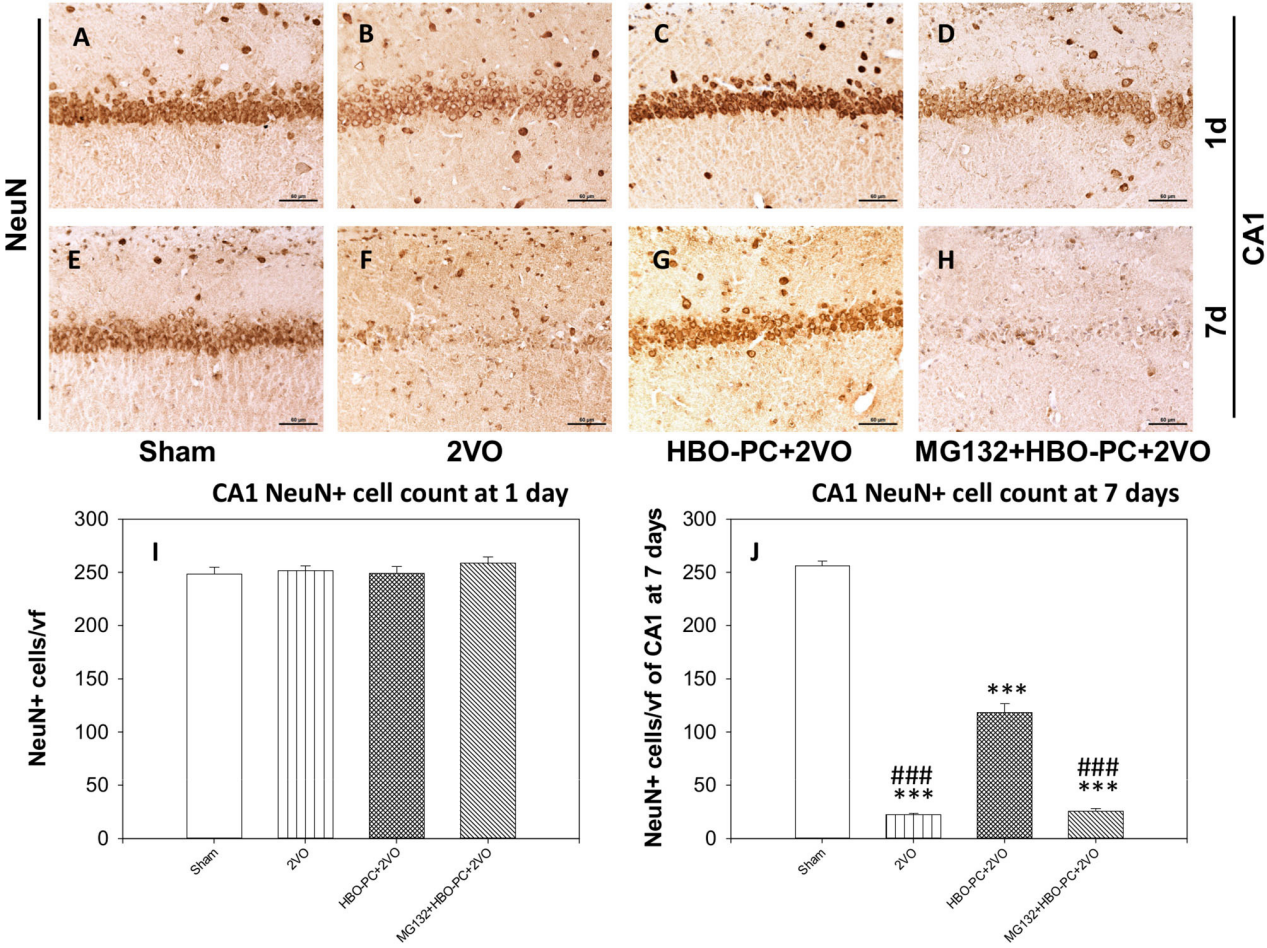
**(A–H)** NeuN immunostaining. Partial sparing of hippocampal CA1 NeuN-expressing cells with HBO-PC is seen. Scale bar = 60 μm. **(I,J)** Numbers of NeuN expressing cells of CA1 at 1 and 7 days post-surgery; ***p < 0.001 vs. sham; ^###^*p* < 0.001 vs. HBO-PC; *n* = 4. HBO-PC, preconditioning with hyperbaric oxygen.

The experimental cerebral ischemia alone tended to weaken brain expression of NeuN at 24 h after 2VO (Figure 3B) as compared to the sham group (Figure 3A). Furthermore, NeuN immunoreactivity was hardly present on the 7th post-ischemic day (Figure 3F) as compared to the sham group (Figure 3E). Expression of NeuN in the preconditioned group appeared stronger than in the non-preconditioned rats on day 1 (Figure 3C) and at 7 days post-ischemia (Figure 3G). It was however faint in the group receiving MG132 and preconditioning at both time points after ischemia (Figures 3D,H). Although cell counting revealed that the numbers of NeuN positive cells were equivalent across experimental groups on day 1 (Figure 3I), on day 7 NeuN positive cells were significantly more numerous in the HBO-PC group as compared to unpreconditioned ischemia and HBO-PC pretreated with a proteasome inhibitor (Figure 3J).

### HBO-PC Attenuates Glial Response to Ischemia in CA1 of the Rat Hippocampus

**Figure 4:**
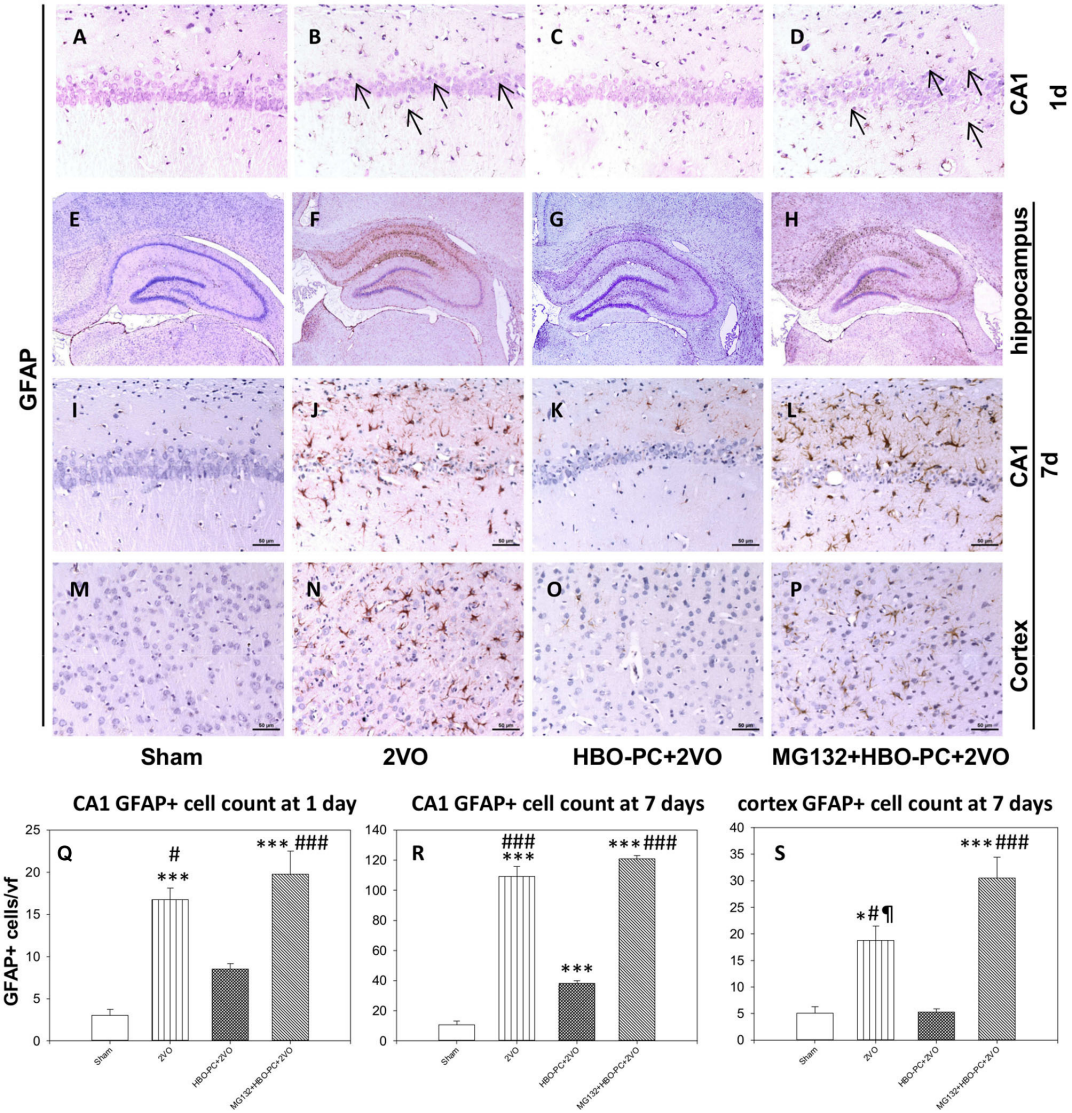
**(A–P)** GFAP immunostaining in the CA1 and cerebral cortex. HBO-PC reduced GFAP immunoreactivity within vulnerable brain structures. In untreated ischemia and MG132+HBO – PC+2VO groups a greater abundance of GFAP positive cells was noted, even on day 1 (arrows) as compared to sham. Scale bar = 50 μm. **(Q–S)** Cell count analysis of GFAP positive cells corroborate differences determined with immunohistochemical observations; *p < 0.05 vs. sham; ***p < 0.001 vs. sham; ^#^*p* < 0.05 vs. HBO-PC + 2VO; ^###^*p* < 0.001 vs. HBO-PC; ^¶^*p* < 0.05 vs. MG132 + HBO-PC + 2VO; *n* = 4. 2VO, two vessel occlusion; HBO-PC, preconditioning with hyperbaric oxygen.

In control brains, no activated GFAP positive astrocytes were seen (Figures 4A,E,I,M). After ischemia, a strong astroglial reaction was localized in the vicinity of neuronal loss within the hippocampus and cerebral cortex especially on day 7 (Figures 4B,F,J,N). Astroglial reaction to ischemia, as determined with GFAP stain, especially around the CA1 sector, was reduced with HBO-PC (Figures 4C,G,K,O). However, the HBO-PC combined with proteasome inhibitor resulted in post-ischemic changes of GFAP expression that were similar to those after the ischemia alone. In this MG132+HBO-PC+2VO group, neuronal loss was associated with strong astrogliosis localized around CA1 and the hilus of the dentate gyrus and in the cerebral cortex especially on day 7 (Figures 4D,H,L,P). The quantitative immunohistochemistry analysis revealed that the number of GFAP positive cells were increased over five-fold after 2VO in CA1 already on day 1, although it was significantly reduced with the preconditioning. Administering MG132 brought back an increased number of GFAP positive cells in CA1 (Figure 4Q). Whereas on the post-ischemic day 7, the number of GFAP positive cells were increased 10-fold in CA1 and four-fold in the cortex and even more so in the groups with combined HBO-PC and MG132 treatment (Figures 4R,S).

### HBO-PC Enhances Brain Expression of 20S Proteasomal Protein

**Figure 5:**
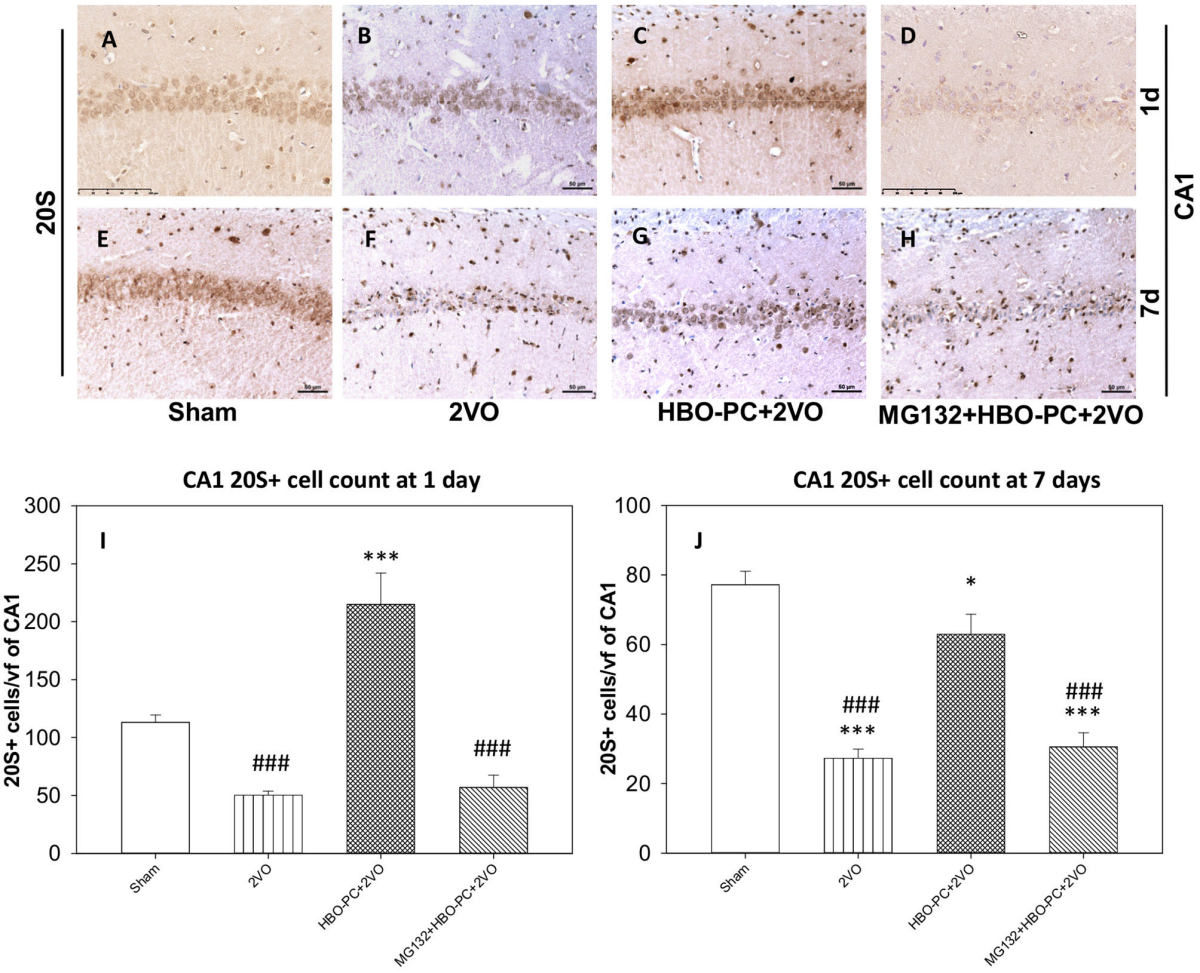
**(A–H)** Brain expression of S20 proteasomal protein. HBO-PC resulted in a preservation of 20S immunoreactivity in the hippocampus post-ischemia. Scale bar = 50 μm **(I,J)** 20S-positive cell count in CA1 at 1 and 7 days; *p < 0.05 vs. sham; ***p < 0.001 vs. sham; ^###^*p* < 0.001 vs. HBO-PC+2VO; *n* = 4. 2VO, two vessel occlusion; HBO-PC, preconditioning with hyperbaric oxygen.

A 20S immunostaining was characterized by a strong expression, while being slightly present in the peripheral cytoplasm of cells in control hippocampi (Figures 5A,E). At 7 days time point after untreated global ischemia, there was a widespread tissue depletion of the 20S immunostaining in the CA1 (Figure 5F), which, in contrast, was fairly well-preserved in surviving neurons after HBO-PC (Figure 5G). The post-ischemic expression of the 20S was weak when HBO-PC and MG132 were combined (Figure 5H). Interestingly, the 20S stain was enhanced in the hippocampus at 24 h after ischemia preceded by HBO-PC (Figure 5C), as compared to non-preconditioned ischemia (Figure 5B) and HBO-PC+MG132 treatment (Figure 5D).

Accordingly at 1 day, 20S positive cells were significantly more numerous in the HBO preconditioned group as compared to all other groups (Figure 5I). CA1 hippocampal cell count at 7 days revealed significant reductions in the numbers of 20S positive cells in the 2VO and proteasome inhibitor groups, as compared to sham, while only slight (although significant) reduction was calculated in the preconditioned group (Figure 5J).

### Bim Cellular Expression Is Reduced in CA1 Sector of the Rat Hippocampus and Cortex With HBO-PC

**Figure 6:**
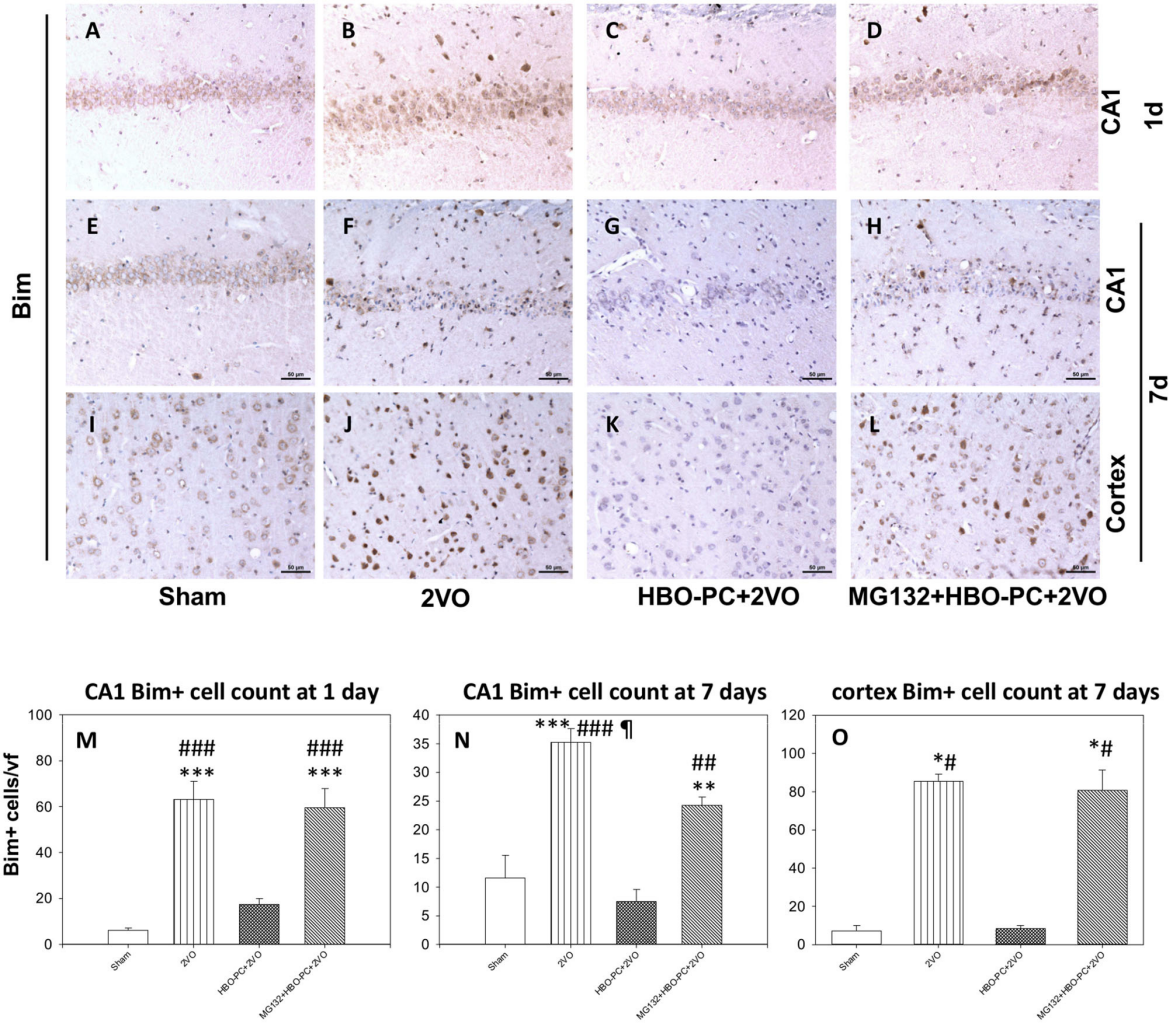
**(A–L)** Bim immunostaining. HBO-PC reduced Bim immunoreactivity post-ischemia while the proteasome inhibitor exerted the opposite effect. Scale bar = 50 μm **(M,N)** Bim-positive cell count in the CA1; **p < 0.01 vs. sham; ***p < 0.001 vs. sham; ^##^*p* < 0.01 vs. HBO-PC + 2VO; ^###^*p* < 0.001 vs. HBO – PC + 2VO; ^¶^*p* < 0.05 vs. MG132 + HBO-PC + 2VO; *n* = 4; ANOVA-SNK. **(O)** Bim-positive cell count in the cerebral cortex; *p < 0.05 vs. sham; ^#^*p* < 0.05 vs. HBO-PC + 2VO; *n* = 4. 2VO, two vessel occlusion; HBO-PC, preconditioning with hyperbaric oxygen.

Rabbit anti-B cell lymphoma-2 interacting mediator of cell death showed an enhanced immunostaining in neurons of CA1 and cerebral cortex after ischemia (Figures 6B,F,J) as compared to that in the sham group (Figures 6A,E,I). A weak Bim expression was found after ischemia preceded by HBO-PC (Figures 6C,G,K). Using MG132 prior to each preconditioning session resulted in the reappearance of Bim tissue expression (Figures 6D,H,L).

Cell count analysis showed an increased number of Bim immunoreactive cells in the brain after ischemia, in the CA1 and cerebral cortex as compared to sham. These increases were prevented in the CA1 and cerebral cortex of animals preconditioned with HBO. MG132 reversed the HBO-PC-induced decrease in numbers of Bim positive cells in the cerebral cortex and partly in CA1 after ischemia (Figures 6M–O).

### Global Cerebral Ischemia Increases p53 Immunoexpression in the Hippocampus

**Figure 7:**
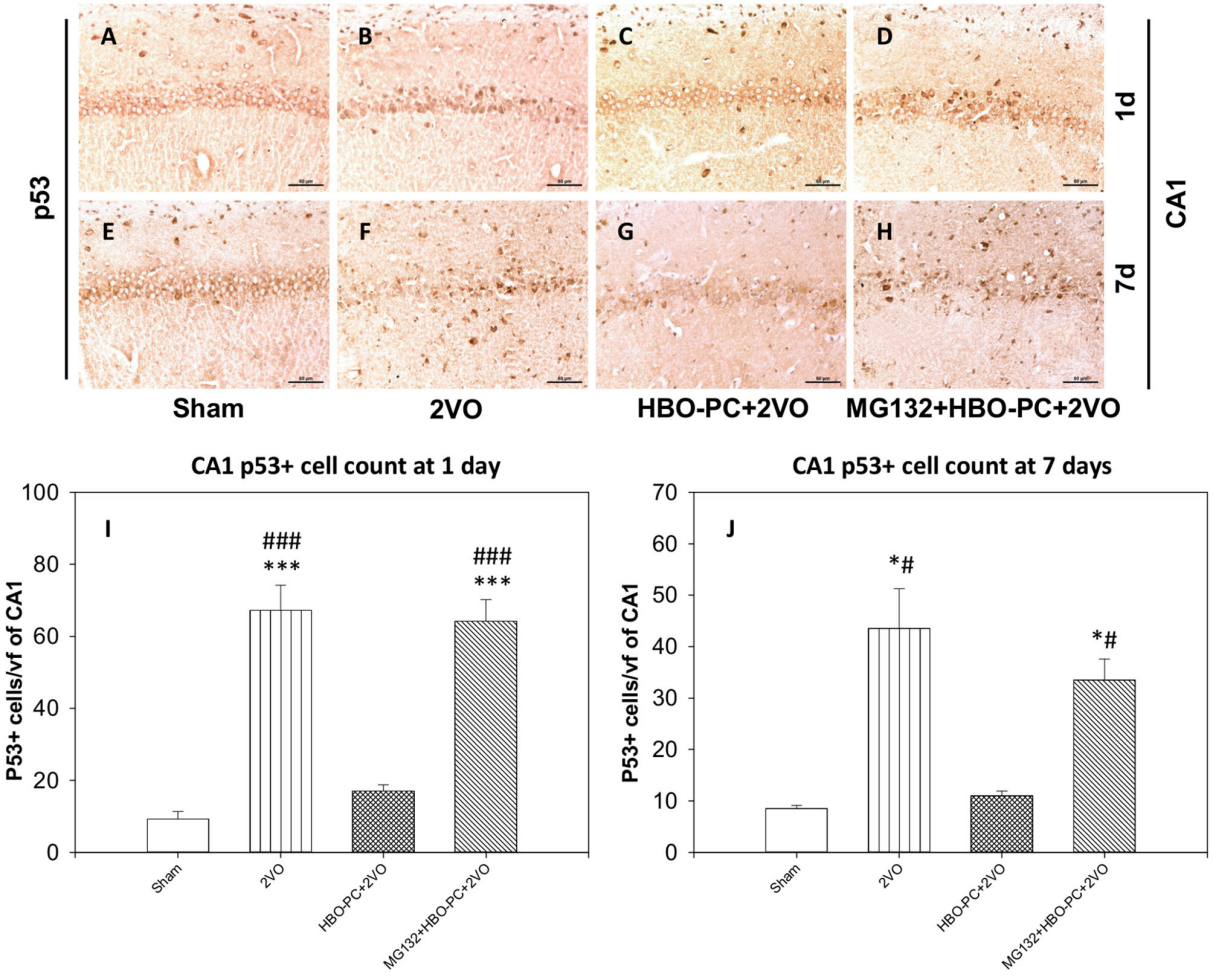
**(A–H)** p53 immunostaining in the hippocampus. The enhanced expression of p53 in the hippocampus was observed early after untreated ischemia. Scale bar = 60 μm **(I)** p53-positive cell count at 1 day. ***p < 0.001 vs. sham; ###*p* < 0.001 vs. HBO-PC+2VO. **(J)** p53-positive cell count at 7 days; *p < 0.05 vs. sham; #*p* < 0.05 vs. HBO-PC+2VO; *n* = 4. 2VO, two vessel occlusion; HBO-PC, preconditioning with hyperbaric oxygen.

The protein of p53 showed predominantly nuclear expression, enhanced already on day 1 after ischemia in the CA1 (Figure 7B) as compared to that in the sham group (Figure 7A). At this time point, only a weak p53 expression was found after ischemia preceded by HBO-PC (Figure 7C). At 7 days post-ischemia, the conspicuous p53 stain colocalized with CA1 injury in the non-preconditioned group (Figure 7F) as compared to sham (Figure 7E); however, p53 stain was weak in the HBO-PC+2VO group (Figure 7G). MG132, administered prior to each preconditioning session, resulted in the reappearance of p53 tissue expression in the CA1 at both time points (Figures 7D,H). On days 1 and 7, numbers of p53 positive CA1 cells increased several-fold post-ischemia, while only less than 1.5-fold in the preconditioned groups as compared to sham. However, in the pretreated with MG132 and preconditioned group, there was again several-fold increases at 1 and 7 days post-ischemia as compared to sham (Figures 7I,J).

### HBO-PC Reduces Post-Ischemic Expression of PUMA in the Cerebral Cortex and CA1 Zone of the Hippocampus

**Figure 8:**
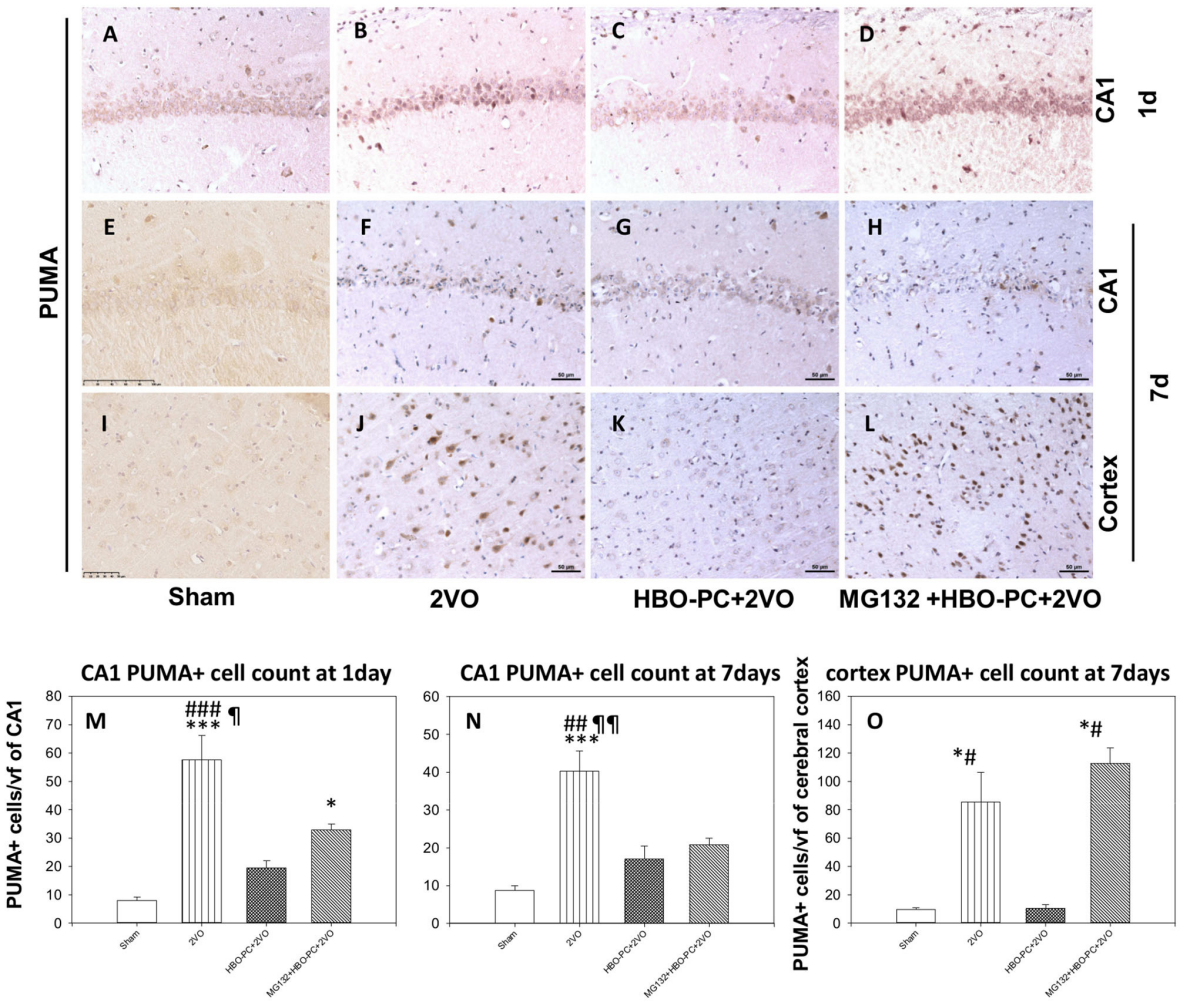
**(A–L)** Brain tissue expression of PUMA. A great abundance of cells expressing PUMA was shown 7 days after ischemia especially in the cerebral cortex, but not in the preconditioned group. Scale bar = 50 μm. **(M,N)** CA1 PUMA immunoreactive cell count. *p < 0.05 vs. sham; ***p < 0.001 vs. sham; ^##^*p* < 0.01 vs. HBO-PC + 2VO; ^###^*p* < 0.001 vs. HBO-PC+2VO; ^¶^*p* < 0.05 vs. MG132+HBO-PC+2VO; ^¶¶^*p* < 0.01 vs. MG132+HBO-PC+2VO; *n* = 4 **(O)** PUMA-positive cell numbers in the cerebral cortex; *p < 0.05 vs. sham; ^#^*p* < 0.05 vs. HBO-PC+2VO; *n* = 4. 2VO, two vessel occlusion; HBO-PC, preconditioning with hyperbaric oxygen; PUMA, p53 upregulated modulator of apoptosis.

Rabbit anti-p53 upregulated modulator of apoptosis showed a weak diffuse neuronal immunostaining in the hippocampus and cerebral cortex in the control group (Figures 8A,E,I). Whereas, after ischemia, there was a strong PUMA immunoreactivity in pyramidal cells of CA1 and in cortical neuronal cells (Figures 8B,F,J). Post-ischemic PUMA immunostaining was weak in the HBO-PC group, especially in the CA1 zone (Figures 8C,G,K). MG132 administration prior to HBO-PC sessions resulted in the reappearance of more potent PUMA expression as compared to the effect of HBO-PC without proteasome inhibitor (Figures 8D,H,L).

Both in CA1 and cerebral cortex, ischemia resulted in the several-fold increased numbers of PUMA positive cells, as compared to sham (Figures 8M–O). In CA1, the preconditioning was associated with insignificant increases of PUMA positive cells, while the addition of MG132 restored a significant increase in the number of PUMA positive cells as compared to control on day 1. In the cerebral cortex, preconditioning resulted in the number of PUMA positive cells being equivalent to control, while the addition of MG132 caused the reappearance of PUMA positive cells in high number with respect to sham.

### Nestin Expression Is Enhanced With the HBO Preconditioning

**Figure 9:**
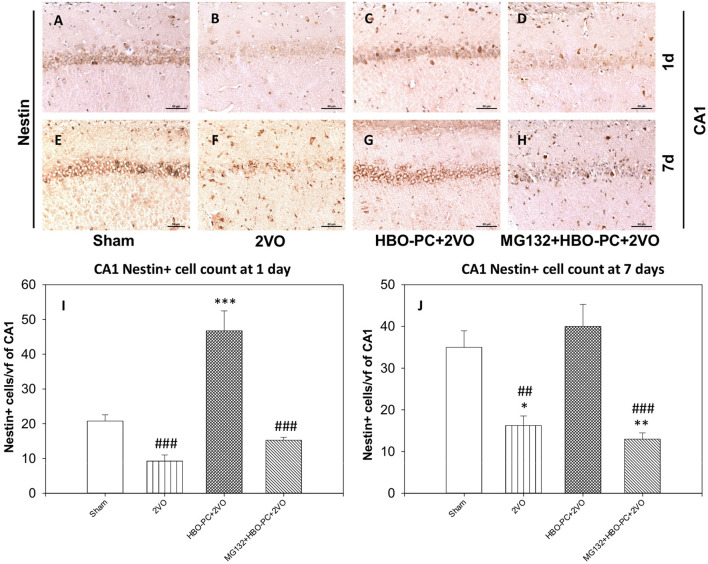
**(A–H)** Nestin immunostaining in rat hippocampus. HBO-PC increases the immunoreactivity of neural stem cells marker nestin. Scale bar = 60 μm. **(I,J)** Numbers of nestin positive CA1 cells are significantly greater in the HBO preconditioned groups as compared to 2VO and MG132+HBO-PC+2VO groups at 1 and 7 days post-ischemia; *p < 0.05 vs. sham; **p < 0.01 vs. sham; ***p < 0.001 vs. sham; ^##^*p* < 0.01 vs. HBO-PC+2VO; ^###^*p* < 0.001 vs. HBO-PC+2VO. 2VO, two vessel occlusion; HBO-PC, preconditioning with hyperbaric oxygen.

Nestin staining showed a depletion throughout the hippocampal sectors on post-ischemic days 1 and 7 (Figures 9B,F) as compared to the staining in the sham group (Figures 9A,E). However, preconditioning enhanced the expression of nestin in the cells of the CA1 on days 1 and 7 after ischemia (Figures 9C,G). MG132 largely reduced the elevating effect of HBO-PC on nestin expression (Figures 9D,H). Cell count revealed that HBO-PC increased the number of nestin positive cells as compared to both sham and 2VO on day 1 after ischemia. In addition, HBO-PC normalized the number of nestin positive cells to the level found in the sham group, thus preventing nestin depletion after untreated ischemia. MG132 administered prior to preconditioning sessions restored numbers of nestin positive cells to the levels equivalent to those in untreated ischemia (Figures 9I,J).

## Discussion

The following are major findings of the present study: HBO preconditioning was effective in reducing CA1 hippocampus cell death and neurobehavioral deficits after global brain ischemia induced by bilateral common carotid artery occlusion and profound hypotensive state. HBO preconditioning enhanced proteasome 20S protein cellular expression and reduced the number of cells expressing cell death proteins, such as Bim, p53, and PUMA. In addition, the abundance of nestin immunopositive cells was greater with the preconditioning. After brain ischemia in the preconditioned rats, the enhanced immunoreactivity of the proteasome was associated with a reduced number of cells expressing apoptotic proteins in the CA1 of the hippocampus and in the cerebral cortex. These findings may provide insight into the mechanism behind HBO-PC-induced neuroprotective effect against neuronal damage and the increased resilience of cells against consequences of the ischemic insult. A significant increase in CA1 cell survival after HBO-PC can be substantial for regaining neurobehavioral functions, especially considering that HBO-PC may additionally activate delayed neurorepair mechanisms, which requires further studies (12).

By utilizing damaged proteins, proteasomes play an important role in the survival of neurons within the brain (13). The expression of 20S proteasome proteins correlates well with the activity of the entire protein-shredding proteasomal complex (14). In response to HBO, greater expression of 20S proteasome protein was observed in this present study. At the same time, the proteins involved in cell death mechanisms were suppressed as was the extent of cell death in the brain. Of those, p53 may trigger the death of cells injured after global brain ischemia (15). The major role in modulating p53 expression is played by proteasomes (16). Proapoptotic protein PUMA is a downstream target of p53 (17). PUMA is also degraded by proteasome, hence upon proteasome dysfunction/inhibition, p53 stabilization, and PUMA-mediated cell death may occur (18). The function of PUMA is to bind and inactivate antiapoptotic members of the Bcl-2 family (19). The involvement of PUMA in the delayed cell death of CA1 hippocampal zone post-ischemia was reported previously (20). Likewise, the pro-apoptotic protein Bcl-2-interacting mediator of cell death (Bim) may mediate cell death upon proteasomal inhibition (21). After global brain ischemia-reperfusion, Bim can be involved in neuronal apoptosis (22). Bim interacts with Bax to translocate to mitochondrial membranes prior to the cytochrome c release and cell death (23).

Nestin is a marker of central nervous system progenitor cells in subventricular zone (SVZ) and subgranular zone (SGZ), the expression of which points toward the potential for cell replacement in the injured brain (24). After brain ischemia, nestin is expressed in astroglia and some neurons whereas, for endothelial, ependymal, and progenitor cells, the expression is seen under normal conditions (25). In response to HBO, nestin expression is enhanced in the neonatal brain with hypoxic-ischemic injury (26). Besides implications for neurogenesis, such findings, have been suggested to indicate the embryonic reversion of the mature cytoskeleton and structural remodeling of the vasculature-associated cells (27, 28).

Interestingly, the link between loss of p53 and facilitation of nestin expression in cerebral tissues has been previously suggested (29). However, the exact mechanisms of interactions between p53 and nestin remain unclear. More importantly, oxidative stress has been reported to enhance nestin expression in other cell systems (30). Previously, an increased abundance of the progenitor cell-specific protein nestin was found in the immature brain subjected to hyperoxia at P6 (31). That nestin expression can be promoted in response of mature neuronal cells to HBO, which is another interesting finding of this present study.

Therapeutically optimal proteasome activation in response to HBO may depend on the regimen of treatment. While a prolonged exposure to hyperoxia may decrease 20S proteasome activity and increase levels of apoptotic proteins (32), short repetitive hyperbaric oxygenation might generate opposite results, leading to a conditioning effect. It has been also shown that modulating pressure conditions may affect proteasome expression as hyperbaric decompression upregulates proteasome subunit beta type-7 in the rat brain (33). Future studies will examine whether and how HBO preferentially targets the apoptotic protein for degradation as suggested by this present study. The putative mechanism could involve distinct proteasome receptors that offer docking positions for ubiquitinated substrates, and numerous cellular ubiquitin ligases that collectively might define different levels toward selective protein degradation (34, 35). This way, cell death proteins might be preferentially targeted for degradation in response to HBO, which however requires further studies in order to substantiate.

Since a depletion of cell death proteins occurs via proteasome, their level can be stabilized post-ischemia due to proteasomal impairment. Hence in non-preconditioned cells, the accumulation of ubiquitinated cell death proteins may occur due to proteasome insufficiency (36). This study also suggests that blocking proteasomal activity by MG132 reversed effects of HBO preconditioning, such as apoptotic protein depletion, cell survival, and neurological improvement. It is rather unlikely that MG132 merely worsens stroke outcomes, hence it would block any therapeutic effects, not just from HBO-PC. Proteasome inhibitors alone, such as MG132 pretreatment, have shown beneficial effects on the stroke outcomes (37, 38). However, in this present study, proteasome inhibitor MG132 in combination with HBO over 5 days prior to ischemia resulted in worse brain and behavioral outcomes compared to stroke animals treated only with HBO. Collectively, these data appear as indicative of blocking the effects of conditioning mechanisms by MG132, which suggests that HBO-PC indeed works via proteasome.

Noticeably though, HBO-PC did not reduce mortality and did not ameliorate sensorimotor impairment related to global cerebral ischemia, as it might not have a sufficient impact on their mechanisms. Mortality and neurologic impairment can be related to overwhelming cell loss upon this model of severe global ischemia, and the difference in cell viability, although statistically significant, was not great enough to alleviate these outcomes upon a 7-day observation period. Further studies of multiple brain regions responsible for these vital functions are needed in this regard. On the other hand, the effect of HBO-PC on neurologic function might emerge at later time intervals post-global ischemia, advocated for evaluation of investigational therapeutics (39).

We chose a subcutaneous injection of MG132 out of several possible routes of administration. Previous studies have shown that proteasome activity inhibition in the rodent brain can be achieved with subcutaneously administered proteasome inhibitors (40). Moreover, a dose of 1 mg/kg was used by earlier authors as a middle-range effective dose of MG132 (41).

Among the limitations of our study is the use of one proteasome inhibitor MG132, which although preferentially targets the proteasome, may have off-target effects. Therefore, future studies might aim to verify that the use of another proteasome inhibitor (e.g., Lactacystin) will result in similar effects. In addition, apart from neurons and astroglia investigated herein, future studies will also examine the role of microglial cells that phagocytose fragmented DNA in the CA1 pyramidal layer after forebrain ischemia and may differentiate into anti-inflammatory M2 phenotype upon hyperbaric oxygenation (42, 43).

## Conclusions

We conclude that HBO-PC-induced brain protection against severe ischemic brain insult appears to involve the proteasome. Thus, previously reported pathways of preconditioning, such as HIF-1α, may depend on and interact with ubiquitin-proteasome system (UPS) (44). Future studies will examine in detail the involvement of all components of the UPS pathway in the mechanisms of HBO-PC-induced neuroprotection. It also remains to be determined whether co-administration of HBO-PC in combination with proteasome modulators may potentiate the beneficial effect of HBO-PC in experimental and clinical studies of cerebral ischemia.

## Data Availability Statement

The raw data supporting the conclusions of this article will be made available by the authors, without undue reservation.

## Ethics Statement

The animal study was reviewed and approved by First Warsaw Local Ethics Committee for Animal Experimentation.

## Author Contributions

RO conceptualized the idea, carried out experiments, performed data analysis, and drafted the manuscript. EP performed experiments and performed data analysis. EM reviewed the study for important intellectual content and edited the manuscript. All authors contributed to the article and approved the submitted version.

## Funding

This work was supported by the Foundation for the Development of Diagnostic and Therapy, Warsaw (Award #11/2020).

## Conflict of Interest

The authors declare that the research was conducted in the absence of any commercial or financial relationships that could be construed as a potential conflict of interest.

## Publisher's Note

All claims expressed in this article are solely those of the authors and do not necessarily represent those of their affiliated organizations, or those of the publisher, the editors and the reviewers. Any product that may be evaluated in this article, or claim that may be made by its manufacturer, is not guaranteed or endorsed by the publisher.
